# Transverse colon volvulus in a 15 year old boy and the review of the literature

**DOI:** 10.1186/1749-7922-5-19

**Published:** 2010-07-02

**Authors:** Goher Rahbour, Abraham Ayantunde, Muhammad Rehan Ullah, Sobia Arshad, Rajab Kerwat

**Affiliations:** 1 Department of General Surgery, Queen Mary's Hospital, Sidcup, DA14 6LT, UK; 2 Department of Orthopaedics, Queen Mary's Hospital, Sidcup, DA14 6LT, UK

## Abstract

We report a rare case of transverse colon volvulus in a fifteen year old boy with a review of the literature. This brings the total number of pediatric cases reported in the English literature to fifteen. This case is unusual in that no aetiological factor has been found, in contrast to the majority of the pediatric cases. Diagnosis can be challenging and the effective management remains controversial. The various radiological imaging modalities are presented. The epidemiology, aetiology, diagnosis and management of transverse colon volvulus are discussed. It is important to highlight this case and those in the literature, as many surgeons may never have seen a single case of transverse colon volvulus. It may therefore not be considered in the differential diagnosis of recurrent intermittent abdominal pain or acute intestinal obstruction.

## Background

This case brings the total number of pediatric transverse colon volvulus reported in the English literature to fifteen. Most pediatric cases have been reported in the United States. Approximately three to five percent of all cases of intestinal obstruction are caused by colonic volvulus [1-4]. The disease is even less common in children. Predisposing factors for transverse colon volvulus in children include mental retardation, dysmotility disorders, lax fixation of the hepatic and splenic flexures, chronic constipation and Hirschsprung's disease [1-7]. There was no predisposing factor in this case unlike the majority which have been reported.

## Case Presentation

**Figure 1:**
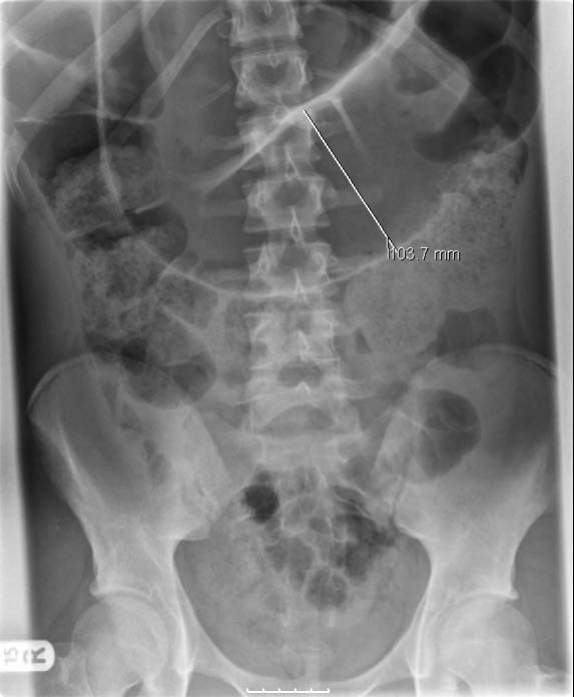
**AXR - Dilated transverse colon**. The descending colon appears collapsed. The distribution of the large bowel dilatation raises the possibility of proximal descending colon obstruction.

**Figure 2:**
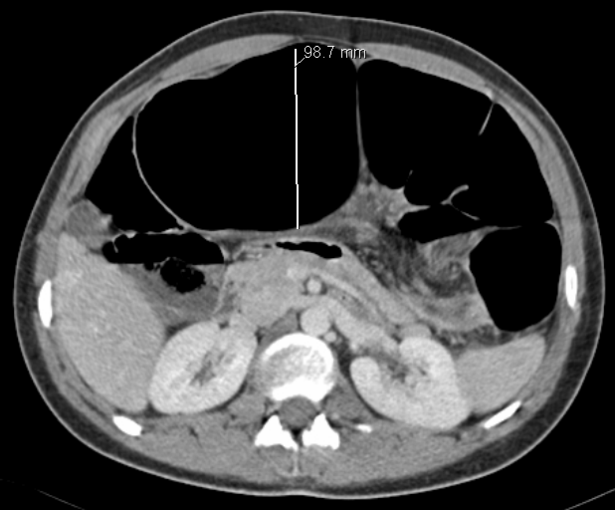
**Abdominal CT provides a differential of a colo-colic intussusception or volvulus**.

**Figure 3:**
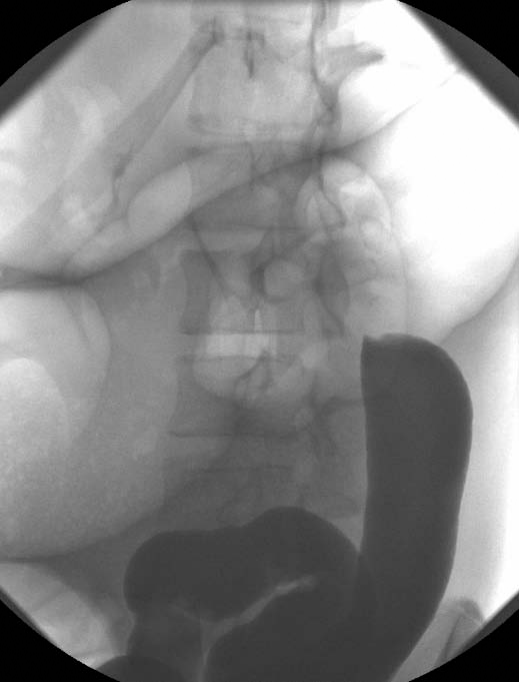
**Water Soluble Contrast Enema (Gastrograffin)**. No therapeutic benefit was achieved. An obstructive lesion in the proximal descending colon is identified. No contrast passed beyond this.

**Figure 4:**
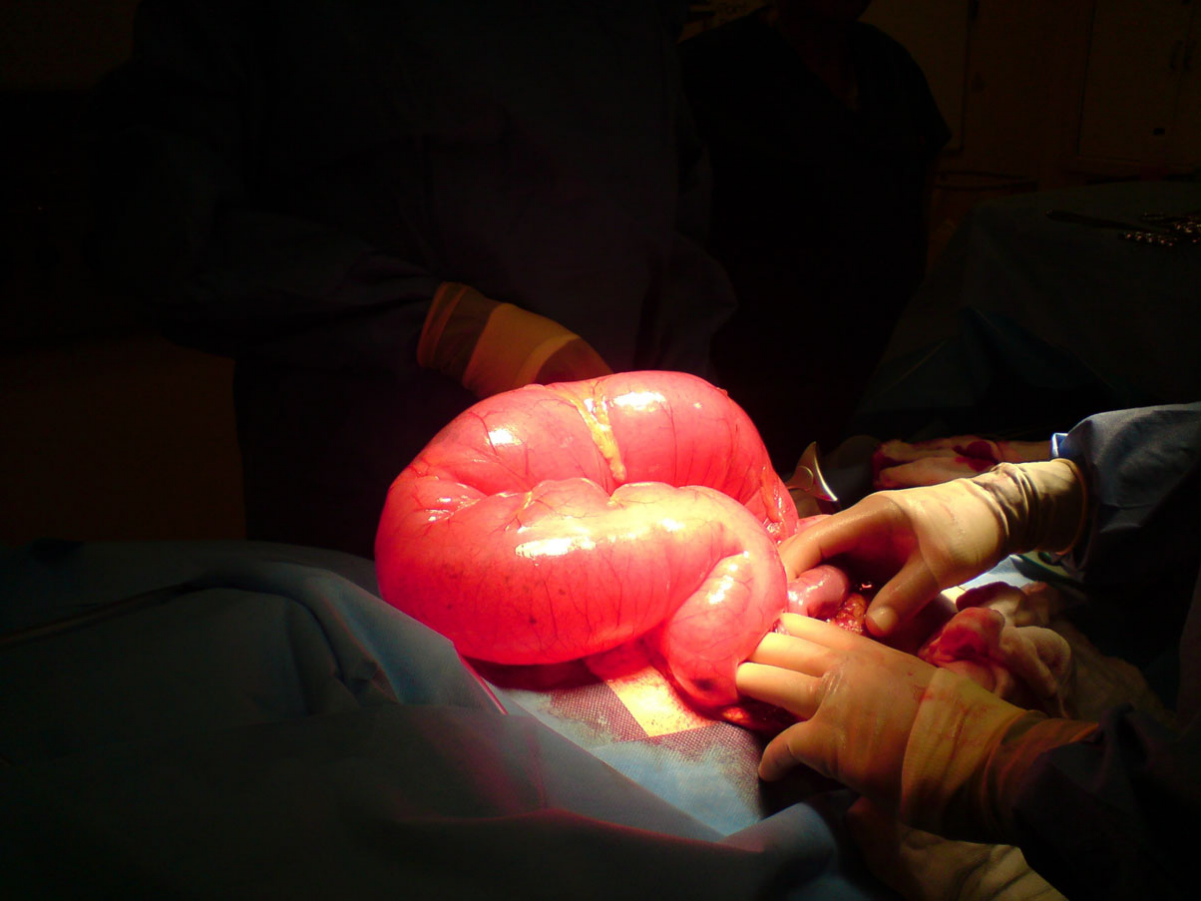
**Transverse Colon Volvulus - Intra operative image of gross large bowel dilatation**.

**Figure 5:**
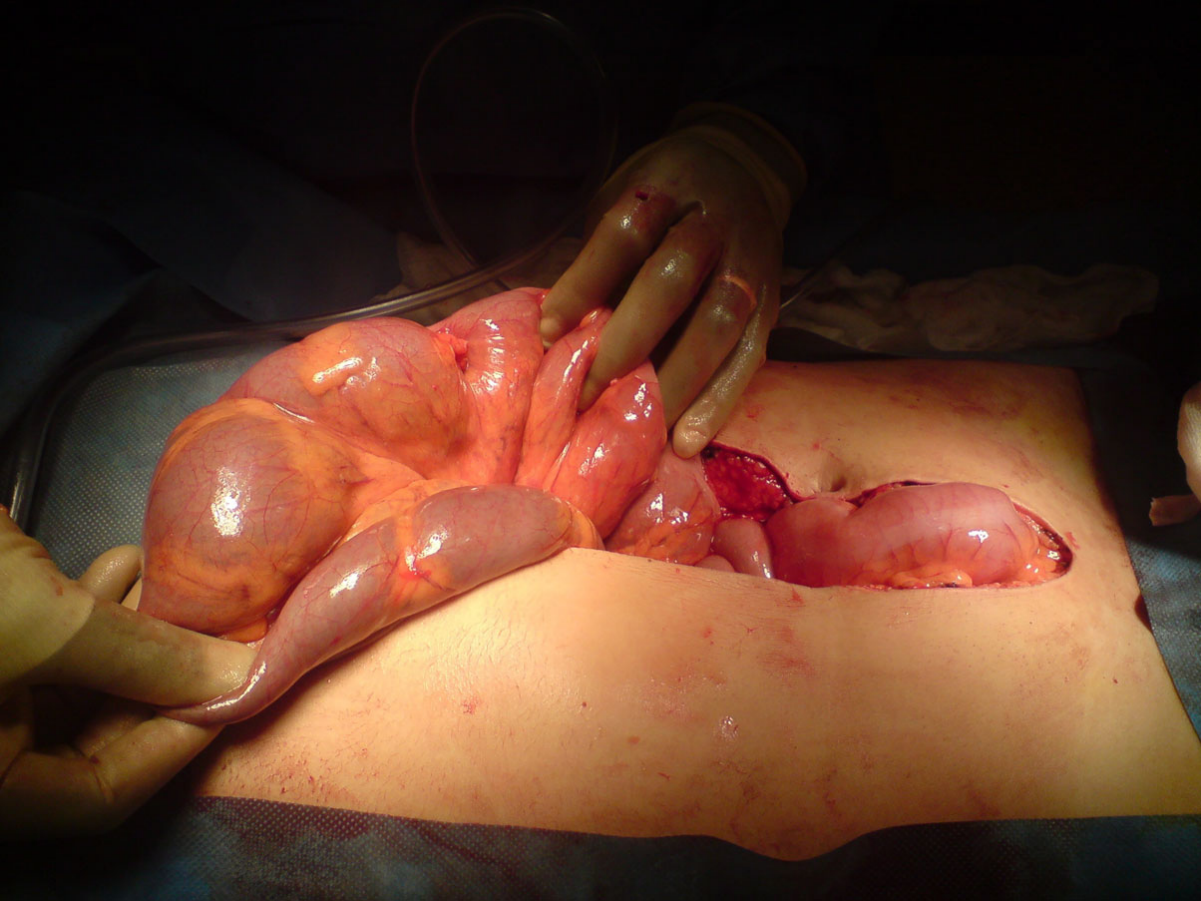
**'Point of twist' was located in the left upper quadrant**.

A fifteen year old boy presented with a three day history of left sided abdominal pain, constipation and vomiting to the pediatricians. Over the preceding year he had several episodes of intermittent abdominal pain. There was no other significant past medical history. Examination revealed mild tenderness in the epigastrium and left side of the abdomen with moderate distension. Blood investigations revealed normal full blood count, urea and electrolytes, liver function tests, and clotting profile. The C-reactive protein (CRP) was four. An abdominal X-ray (AXR) [Fig. 1] revealed a dilated transverse colon. The distribution of the large bowel dilatation should have raised the possibility of proximal descending colon obstruction. However a computer tomography scan (CT) [Fig. 2] was organised. This revealed dilatation of the proximal transverse colon with a cut-off near the splenic flexure. The appearance was suggestive of a colo-colic intussusception or a volvulus. A surgical review was sought following which a water soluble gastrografin enema was performed for both a therapeutic and diagnostic purpose. This highlighted an obstructive lesion in the proximal descending colon [Fig 3]. No contrast passed beyond this point, and the intended therapeutic benefit was not achieved with the procedure. An emergency laparotomy was performed for large bowel obstruction. Intra operative findings were of a transverse colon volvulus [Fig 4] rotated in a three hundred and sixty degrees clockwise direction. The point of twist was found in left upper quadrant [Fig 5], in keeping with the pre operative imaging. The transverse colon was mobilised, resected at the splenic flexure and just short of the hepatic flexure. A side to side anastomosis was performed for establishing bowel continuity because of significant disparity in the size of the obstructed proximal and collapsed distal colon to the site of the volvulus. A loop defunctioning ileostomy was fashioned.

A prolonged post operative ileus developed. This was partially attributed to initial difficulty in adequate pain control with the use of opiate analgesia. A gradually rising CRP to four hundred and nine over the course of a week led to a CT scan being performed. This demonstrated no free fluid or evidence of an anastomotic leak. With the development of sepsis of unknown origin, a decision was taken for a further re-look laparotomy eight days after the initial laparotomy. There was no free fluid in the abdominal cavity and the anastomosis was intact. Discharge from hospital was twenty three days following admission.

Histology demonstrated the large bowel to have continuous mucosal architectural abnormality including crypt distortion. There was associated marked thickening of the muscularis mucosa. The luminal surface was unremarkable. The lamina propria showed widespread haemorrhage with preserved cellularity gradient. No acute inflammation, infarction, granulomas, dysplasia, malignancy, vascular abnormality was seen. The bowel was ganglionated throughout. There was no evidence of chronic idiopathic inflammatory bowel disease. Lymph nodes showed marked oedema with blood engorgement in the sinuses. Both resection margins of the specimen revealed normal bowel architecture and hence the entire affected segment of the transverse colon had been resected. Histologically, the appearances were consistent with a sub acute progressive transverse colon volvulus.

**Figure 6:**
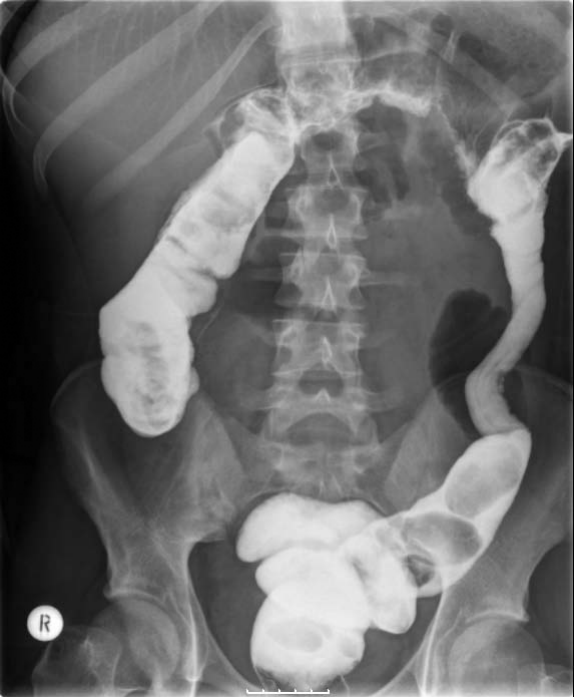
**Water Soluble Contrast Enema - Contrast was introduced per rectum**. This was seen to flow freely to the right side of the abdomen within the bowel. No extravasation of contrast or stricture was demonstrated.

The child was readmitted on three occasions over the next three months with recurrent adhesive small bowel obstruction which was managed conservatively. A water soluble contrast enema [Fig 6] demonstrated contrast to flow freely to the right side of the abdomen within the bowel. He subsequently underwent a laparoscopic adhesiolysis and closure of the ileostomy. Slow progress and the development of ileus necessitated his transfer to a regional pediatric surgical unit for subsequent management. Multiple rectal biopsies were taken, and these showed the presence of ganglion cells and the absence of thickened nerves. This combination of histopathological findings did not support a diagnosis of Hirschsprung's disease.

We conclude that neither the histopathology from the gross specimen nor the rectal biopsies is in keeping with a dysmotility disorder and hence this cannot explain the delayed recovery and prolonged ileus.

## Discussion

There are only fifteen cases of paediatric transverse colonic volvulus so far in the literature including this present case (Table 1). Of all cases there was seven male and seven female children. One case had no sex documented. The mean age was ten years. Presenting symptoms included abdominal distension: fifteen, vomiting: eleven, constipation: seven. The following past medical history were indicated in the patients; mental retardation: five, chronic constipation: five, previous Hirschprung's disease: one. Management included manual detorsion without any further procedure: five, bowel resection: nine, colostomy: five, ileostomy: one. Two children passed away (respiratory infection and aspiration). Transverse colon volvulus was found to be in a clockwise direction in six cases, and anticlockwise direction in three. The remaining cases had no documentation to the direction of volvulus.

**Table 1:** Cases of pediatric transverse colon volvulus in the literature [2,3,5,8,9]

No.	Author (et al)	Year	Age	Sex	Presentation	Past medical history	Degree and direction of rotation	Management
1	Massot	1965	2	F	distension	nil	360° anti- clockwise	Detorsion
2	Cuderman	1971	10	F	vomiting distension	mental retardation, chronic constipation	clockwise	Colectomy, double barrel colostomy
3	Howell	1976	4	F	vomiting distension	chronic constipation	anti- clockwise	Detorsion, mesocolon resection, colostomy
4	Howell	1976	16	F	vomiting constipation distension	recurrent episodes	N/A	Transverse colon resection, colostomy
5	Eisenstat	1977	15	F	vomiting distension	mental retardation	N/A	Resection, colostomy. Aspirated: died 4^th ^day post operative
6	Dadoo	1977	12	M	constipation distension	recent severe diarrhoea	360° anti- clockwise	Detorsion. Elective resection
7	Neilson	1990	11	M	vomiting constipation distension	hirschprung's, chronic constipation	360° clockwise	Detorsion, colostomy
8	Mindelzun	1991	15	M	vomiting distension	mental retardation, chronic constipation	N/A	Detorsion
9	Mellor	1994	2	N/A	vomiting distension	nil	N/A	Detorsion, resection of transverse colon
10	Mercado-Deare	1995	7	M	vomiting distension	mental retardation, myotonic dystrophy, hydrocephalus	360°	Detorsion
11	Houshian	1998	9	F	vomiting constipation distenstion	recurrent episodes	720° clockwise	Detorsion
12	Samuel	2000	5	M	constipation distention	cerebral palsy	N/A	Resection with primary anastomosis
13	Jornet	2003	12	M	distension	nil	180°	Detorsion
14	Liolios	2003	10	F	vomiting constipation distension	trisomy 13, mental retardation, chronic constipation	360° clockwise	Detorsion. Extended right hemi. Died after 28 days: chest infection
15	Rahbour	2010	15	M	vomiting constipation distension	nil	360° clockwise	Transeverse colon resection, loop ileostomy

The aetiologies of transverse colon volvulus may be grouped as mechanical, physiological, and congenital [1-4]. Mechanical causes include: previous volvulus of the transverse or sigmoid colon, distal colonic obstruction, adhesions, malposition of the colon following previous surgery, mobility of the right colon, inflammatory strictures, and carcinoma [1-4]. Twisting usually occurs along the mesenteric axis of the bowel, resulting in venous obstruction and eventually arterial compromise [4]. Volvulus is favoured by elongation of the colon, chronic constipation, or by anatomical defects in the normal liver and colon attachments [5]. Thirty three to thirty five percent of children with volvulus of the transverse colon appear to have had a history of chronic constipation [3], which is either idiopathic or secondary to Hirschprung's disease [3,6,7], mental retardation or myotonic dystrophy. Children with mental retardation will tend to have abnormal and irregular bowel function. Chronic constipation can promote elongation and chronic redundancy of the transverse colon.

The two properties essential to the formation of a volvulus are redundancy and non-fixation. The ascending and descending segments of the colon are fixed, but the sigmoid colon, caecum, and transverse colon are mobile within the peritoneum, tethered by their mesentery. This mobility allows volvulus to occur at these locations. Redundancy of any of these segments further enables the formation of a volvulus [4]. The literature describes two forms of presentation; acute fulminating and subacute progressive. Patients with the acute fulminating type of presentation typically have a sudden onset of severe abdominal pain, rebound tenderness, vomiting, little distension, and rapid clinical deterioration. Bowel sounds are initially hyperactive but may later become absent [3,4]. The acute form presents in sixty percent of children [3]. Subacute progressive transverse volvulus is associated with massive abdominal distension in the setting of mild abdominal pain without rebound tenderness and little or no nausea or vomiting [4]. Our case was clinically of the subacute presentation, and this was correlated with the histological findings.

A transverse colon volvulus does not have the same classically recognisable radiographic features as sigmoid and caecal volvulus. The gold standard of diagnosis is a contrast enhanced plain film which reveals the 'birds beak' phenomenon characteristic of any volvulus. The abdominal film may reveal a large bowel obstruction with proximal colonic distension, two long air-fluid levels and a 'U-shaped' loop with the apex pointing away from the point of torsion of the colon (bent inner tube appearance) [3].

Whereas sigmoid volvulus can often be decompressed by sigmoidoscopy or colonoscopy, transverse colon volvulus must be surgically detorsed [1]. The choice of surgical approach in children is a matter of debate. Avoiding an aggressive intervention such as partial colectomy may minimise post surgical complications, and this was the choice from our decision making [5]. Surgical options include: detorsion alone, detorsion with colopexy, resection with primary anastomosis, or resection with colostomy or ileostomy and mucous fistula. Both detorsion and detorsion with colopexy have a higher rate of recurrence than resection [1,2,4]. Resection with or without primary anastomosis is the treatment of choice for transverse colon volvulus to prevent recurrence [1,4].

## Conclusion

In conclusion transverse colon volvulus is rare, and further more so in the pediatric group. Diagnosis can be challenging and the effective management remains controversial. Many surgeons may never have seen a single case of transverse colon volvulus, and it therefore may not be considered in the differential diagnosis of recurrent intermittent abdominal pain or acute intestinal obstruction. This case highlights that even following repeat biopsies, histology may be normal and hence no identifiable cause to the disease pathology is revealed. Hence this can further complicate the management process in an already unusual and rare case.

## Consent

Written informed consent was obtained from the patient for publication of this case report. A copy of the written consent is available for review by the Editor-in-Chief of this journal.

## Competing interests

The authors declare that they have no competing interests.

## Authors' contributions

All authors were actively involved in the preoperative and postoperative care of the patient. GR performed the literature review drafted the paper and revised the manuscript. MU and SA did literature search and acquired the figures. AA and RK performed the surgery, provided the intraoperative images and revised the manuscript.

All authors read and approved the final manuscript.
